# High mortality due to snakebites in French Guiana: Time has come to re-evaluate medical management protocols

**DOI:** 10.1371/journal.pntd.0006482

**Published:** 2018-07-19

**Authors:** Rémi Mutricy, Xavier Heckmann, Maylis Douine, Christian Marty, Anne Jolivet, Véronique Lambert, Frédérique Perotti, David Boels, Sébastien Larréché, Jean-Philippe Chippaux, Mathieu Nacher, Loïc Epelboin

**Affiliations:** 1 CIC Inserm 1424 Antilles Guyane, Centre Hospitalier Andrée Rosemon, Cayenne, French Guiana; 2 Emergency Department, Centre Hospitalier Andrée Rosemon, Cayenne, French Guiana; 3 Emergency Department, Centre Hospitalier de l’Ouest Guyanais, Saint-Laurent-du-Maroni, French Guiana; 4 Ecosystèmes Amazoniens et Pathologie Tropicale, Université de Guyane, Cayenne, French Guiana; 5 French Red Cross, Cayenne, French Guiana; 6 Department of Public Health, Centre Hospitalier de l'Ouest Guyanais, Saint-Laurent-du-Maroni, French Guiana; 7 Department of Obstetrics and Gynecology, Centre Hospitalier de l'Ouest Guyanais, Saint-Laurent-du-Maroni, French Guiana; 8 Pharmacy, Centre Hospitalier de l’Ouest Guyanais, Saint-Laurent-du-Maroni, French Guiana; 9 Poison Control Center, Angers University Hospital, Angers, France; 10 Medical Biology Department, Hôpital d'Instruction Des Armées Bégin, Paris, France; 11 CERPAGE, Faculté des Sciences de la Santé, Université d'Abomey-Calavi, Cotonou, Bénin; 12 Mère et enfant face aux infections tropicales and PRES Sorbonne Paris Cité, Université Paris Descartes, Faculté de Pharmacie, Paris, France; 13 Infectious and Tropical Diseases Department, Centre Hospitalier Andrée Rosemon, Cayenne, French Guiana; College of Health Sciences, Bayero University Kano, NIGERIA

## Context

On January 15, 2017, a 39-year-old man died from uncontrollable hemorrhagic shock after being bitten by an unidentified venomous snake, presumably *Bothrops atrox*, in Cayenne, the main city of French Guiana.

French Guiana is located in the Amazonian area on the northeastern coast of South America between Suriname and Amapá, Brazil. This event, relayed by regional and national media, took on an unexpected dimension amplified by political tensions at that time. The nonuse of antivenom (AV) in the Cayenne hospital was the focus of critics and presented as a reflection of mainland France’s neglect of French Guiana. We discuss here the factors that led to this clinical situation and the resulting challenges.

Among the hundred species of snakes identified in French Guiana, less than 15 are potentially dangerous for humans (6 species of Viperidae and 6 species of Elapidae) [1, 2]. The Viperidae are the most frequently responsible for attacks, mainly *B*. *atrox*, followed by *B*. *brazili*, *B*. *bilineatus*, and *Lachesis muta*. Bites from other species have been described but remain rare: *Crotalus durissus* is confined to the coastal savannahs, and bites by *Micrurus* sp.—the only genus belonging to elapid family—are rare. However, it is difficult to have a precise estimation of the relative proportion of species incriminated in snakebites, as snake identification is possible in less than 5% of the cases; in most cases, patients consult at the emergency ward without the specimen [3]. In recent publications in Manaus, Amazonas, Brazil, genus identification based on clinical and epidemiological criteria was reported in 91% of cases [4].

The aim of this editorial is to synthesize the information available and to make clear proposals to the French health authorities to improve the management of snakebites in French Guiana.

## Morbidity and mortality due to snakebites in French Guiana

Until 2016, morbidity and mortality linked to ophidian envenomation were considered to be low in French Guiana. However, this impression was not supported by any recent data since no study had been published after 1984 [2] and case notification is not mandatory.

**Fig 1:**
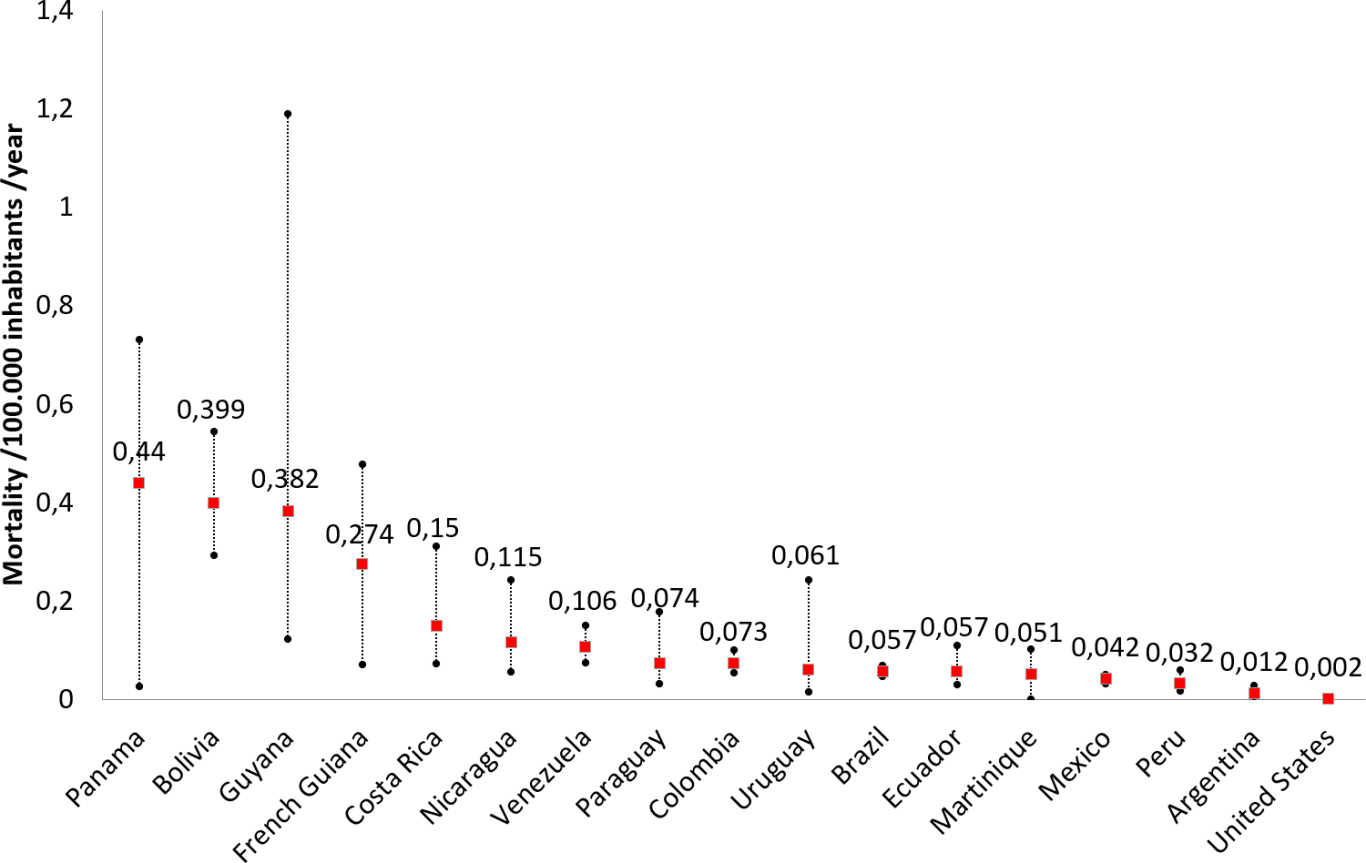
Mortality rates in various countries of the Americas with updated data in French Guiana.

We recently published a retrospective study about morbidity and mortality of snakebites in patients seen in the Cayenne hospital [3]. The study included 425 patients who consulted for snakebites between 2007 and 2015. One-third (142/425) were asymptomatic bites. Of the 283 actual envenomations, 43 (10.1% of total bites and 15.8% of clinical envenomations) were severe, and 4 resulted in death (0.9% of total bites and 1.4% of real envenomations). To have an overview of the situation in all of French Guiana, we compiled data from all health facilities in French Guiana (decentralized prevention and primary healthcare centers spread in remote areas and the hospitals of Saint-Laurent-du-Maroni and Kourou) until December 31st, 2017. We calculated the annual incidence rate using census data (www.insee.fr). During the study period (January 1, 2007, to December 31, 2017), Mutricy and colleagues [3] reported 4 deaths in the Cayenne hospital between 2007 and 2015, which were added to 3 other deaths in other parts of the territory. Thus, the snakebite mortality was 0.274 per 100,000 people per year (95% confident interval 0.071–0.477). However, these figures could be underestimated because either snakebites were not notified and/or the denominator remains imprecise due to a large population of illegal gold miners living in the Amazonian forest, some of whom may have died from snakebites without consulting the healthcare system. We compared the mortality rate with recent estimates from other countries or territories in the Americas [5]. As a consequence, specific mortality in French Guiana appears to be the fourth highest in Latin America, just after Panama, Bolivia, and Guyana (Fig 1).

Since 1984 and the publication relating 2 deaths during the 1982–1983 study [2], there has been no official or published data concerning deaths by snakebites. The only available data appeared in a 2007 doctoral thesis on acute intoxications in the Cayenne hospital that reported only 4 deaths out of 125 ophidian envenomations treated in the intensive care unit (ICU) of the hospital of Cayenne between 1980 and 2007 [6]. Although not isolated to the Americas [5], the apparent rise of mortality by snakebites in French Guiana raises questions. The first explanation may be the rapidly growing population (67,000 inhabitants in 1980, 252,000 in 2014, and 574,000 estimated in 2040). Other reasons may include improved case reporting; changes in behavior, with patients seeing a doctor in the hospital rather than traditional healers; a wildlife closer to the population; increase of illegal gold mining; and lack of access to specialized healthcare, including AVs until 2017.

## Nonuse of antivenom in French Guiana: Time to change?

Although *Micrurus* sp. and *C*. *durissus* show life-threatening neurological toxicity, their bites are infrequent and do not appear to be the priority for an AV. However, *Bothrops* sp. and *L*. *muta* may cause local and systemic disorders: pain, edema, tissue necrosis, renal injury, and hemorrhagic syndrome. The latter involves different mechanisms, such as vascular injury, platelet disorders (mediated by phospholipases A_2_, serine proteinases, or metalloproteinases), consumption coagulopathy induced by factor activators, and fibrino(geno)lysis caused by thrombin-like enzymes, resulting in complications ranging from simple local bleeding to uncontrollable systemic hemorrhages, as was the case for the man who died in January 2017. AV, the only etiological treatment theoretically effective against envenomation, should be active on these clinical conditions. The goal is to promote the clearance of the venom from the vascular compartment.

The AV was not used for several reasons (the arguments are listed in order of relevance): first, the presupposed risk of frequent and severe adverse events due to AVs administration; second, the alleged rarity of the complications and deaths following snakebites; third, the absence of randomized controlled clinical trials demonstrating the efficacy of AV for native snakebites [7]. While randomized trials of AV versus placebo are unethical, studies could compare either 2 different AVs or various doses of the same AV. However, there remains a reasonable doubt regarding the effectiveness of polyvalent AVs marketed in Latin America against the venoms of Guianese snake species (Table 1). There is no production of AVs using venoms from French Guiana snakes, and the venom composition is known to differ from one region to another [8, 9]. Anyway, the demonstration of the effectiveness of an AV in a given territory does not guarantee the effectiveness in others places, even close, as studies have been performed in South America, specifically in Brazil, Colombia and Ecuador [10–12], comparing different national AVs, which showed efficacy of AV therapy in patients submitted to treatment with AVs manufactured with venoms from no Guianese species. Whatever the possible methodological deficiencies, application of the results of any study is questionable for other species or subspecies. A clinical study should confirm the efficacy of eligible AV in French Guiana.

**Table 1:** 2010 WHO list of recommended antivenoms on the WHO database (http://apps.who.int/bloodproducts/snakeantivenoms/database/ -> France(Guiana).

Venomous snake species	Number of possible AVs recommended by WHO	Country
*Bothrops atrox*	16	Brazil, Peru, Ecuador, Colombia
*B*. *bilineatus*	0	
*B*. *brazili*	1 (Antibotropicopolivalente)	Peru
*Lachesis muta*	Antivipmyn TRI but also 5 others, including polyvalents	Brazil, Colombia
*Crotalus durissus*	14	many South American countries
*Micrurus* spp.	3	

**Abbreviation: AV, antivenom**.

Fourth, the high cost of immunotherapy and the poor AV stability under a tropical climate hampered its implementation.

An AV was used in French Guiana during the 1980s. Several arguments had led to the nonuse of AVs in Cayenne Hospital. Finally, although tolerance of AV remains an issue,—the side effects ranging from mild allergic reactions to anaphylactic shock—recent improvements in AV manufacturing allow significant reduction in both their incidence and severity, which can be easily controlled by symptomatic treatment. In 2014, the medical community at Saint-Laurent-du-Maroni Hospital chose a different strategy and decided to use AV for severe snakebites. The main reason for this was the distance from the ICU in Cayenne Hospital (250 km away). After multiple consultations, Antivipmyn TRI was chosen because it is the only AV authorized in French Guiana by the French National Agency for the Safety of Medicines and Health Products (ANSM) and because it fulfills WHO recommendations [13]. A preclinical study, testing several marketed and experimental AVs, showed good results in terms of neutralization of venoms from French Guiana snakes [14]. A prepost retrospective study was recently conducted comparing 28 snakebites treated with AV to 35 non-AV snakebites in the Saint-Laurent-du-Maroni hospital [15]. This study did not demonstrate any benefit of AV on biological coagulation parameters. However, these results should be considered with caution due to the small sample size. The lack of AV efficacy in Saint-Laurent-du-Maroni could be related to an insufficient dose.

First, as shown above, snakebite morbidity and mortality in French Guiana are not trivial.

Second, although many factors influence the variability of the venoms [16], some experimental and clinical studies showed that polyvalent AVs exhibit quite good paraspecificity and cover most of Amazonian species [10, 17]. Indeed, the main species found in Guiana (mainly the 3 *Bothrops* sp. and *Lachesis muta*) are also public health concerns in neighboring countries, and some (Brazil and Colombia) produce high-quality AVs with good efficacy results.

Third, improvements of AV manufacturing (purification of IgG in all marketed AV, enzymatic digestion of antibodies in most marketed AV, and lack of chemical preservative in lyophilized AV) has made the AV much safer and has dramatically reduced adverse events [18].

Fourth, the cost of in-hospital medical treatments is high, especially intensive care admissions, and several studies showed that AVs significantly reduce the risk of complications and hospitalization duration and, consequently, costs [17].

Furthermore, WHO recommends the use of AV throughout the world [13].

Presently, only one AV is authorized by French regulatory authorities. Which AV would ultimately be the best choice for French Guiana is not clear yet. It is therefore necessary to test AVs used in neighboring countries, first in preclinical studies, then in clinical trials. The eligible AVs will be those fitting both the selection criteria defined by WHO [13] and the French ANSM.

Until recently, there was no common strategy in French Guiana. An international symposium held in September 2017 intended to build a treatment consensus, enabling as many patients as possible to benefit from AVs, and to set up surveillance and pharmacovigilance systems. Overall, all agreed on the need to use AV therapy and a common protocol using a higher dose in patients with severe envenomations was validated for the three main hospitals.

## Conclusion

The snakebite mortality rate in French Guiana seems high and deserves attention. Given these new data and improvements in AV safety, the policy of snakebite care has been re-evaluated in our territory, and AV is now used in the two main hospitals of the territory. Although the effectiveness of available AVs has not yet been proven with local species, it is likely that their use will prevent some deaths and severe complications. Conversely, an AV including Guianese species could also benefit neighboring countries because of paraspecifities that could increase their efficiency. As we start in this direction, there are still some aspects that need clarification and vigilance to optimize patient management: preclinical studies of potentially relevant AVs should be performed; the number of injected doses should be optimized; ELISA or molecular biology should be used to identify snake species and to quantify venom concentrations before and after AV administration; treatment protocols should be scaled up to remote areas; despite marked improvements in tolerance, adverse events should be rigorously monitored; and, finally, health authorities should remove the strict, France-centered regulatory barriers that presently forbid the use of most AVs produced in Latin America. They should approve the use of eligible AVs through the introduction of new Temporary Utilization Authorization (ATU) and incite manufacturers to involve French Guiana venoms in their products to be suitable for French Guiana.

## References

[1] UICN France, MNHN, GEPOG, Kwata, Biotope, Hydreco, et al La Liste rouge des espèces menacées en France—Faune vertébrée de Guyane. Paris, France: 2017 978-2-918105-65-7.

[2] Chippaux JP, Galtier J, Lefait JF. Epidémiologie des envenimations en Guyane Française. Bull Soc Pathol Exot Filiales. 1984;77(2):206–15. Epub 1984/03/01. . 6722970

[3] Mutricy R, Egmann G, Marty C, Houcke S, Adenis A, Douine M, et al Predictors of complications of snake envenomation in Cayenne, French Guiana, 2007–2015. Intensive Care Med. 2017 Epub 2017/09/08. 10.1007/s00134-017-4929-3 . 28879463

[4] Feitosa EL, Sampaio VS, Salinas JL, Queiroz AM, da Silva IM, Gomes AA, et al Older Age and Time to Medical Assistance Are Associated with Severity and Mortality of Snakebites in the Brazilian Amazon: A Case-Control Study. PLoS One. 2015;10(7):e0132237 Epub 2015/07/15. 10.1371/journal.pone.0132237 ; PubMed Central PMCID: PMCPmc4500501. 26168155 PMC4500501

[5] Chippaux JP. Incidence and mortality due to snakebite in the Americas. PLoS Negl Trop Dis. 2017;11(6):e0005662 Epub 2017/06/22. 10.1371/journal.pntd.0005662 ; PubMed Central PMCID: PMCPmc5495519. 28636631 PMC5495519

[6] Mayence C. Les intoxications aiguës en Guyane française Enquête rétrospective descriptive sur l'année 2005 au SAMU de Guyane. Nantes, France: Université de Nantes; 2007.

[7] Maduwage K, Buckley NA, de Silva HJ, Lalloo DG, Isbister GK. Snake antivenom for snake venom induced consumption coagulopathy. Cochrane Database Syst Rev. 2015;(6):CD011428. Epub 2015/06/11. 10.1002/14651858.CD011428.pub2 [doi]. . 26058967 PMC11103661

[8] Calvete JJ, Sanz L, Perez A, Borges A, Vargas AM, Lomonte B, et al Snake population venomics and antivenomics of Bothrops atrox: Paedomorphism along its transamazonian dispersal and implications of geographic venom variability on snakebite management. J Proteomics. 2011;74(4):510–27. Epub 2011/02/01. 10.1016/j.jprot.2011.01.003 [doi]. . 21278006

[9] Nunez V, Cid P, Sanz L, De La Torre P, Angulo Y, Lomonte B, et al Snake venomics and antivenomics of Bothrops atrox venoms from Colombia and the Amazon regions of Brazil, Peru and Ecuador suggest the occurrence of geographic variation of venom phenotype by a trend towards paedomorphism. J Proteomics. 2009;73(1):57–78. Epub 2009/08/12. 10.1016/j.jprot.2009.07.013 [doi]. . 19665598

[10] Smalligan R, Cole J, Brito N, Laing GD, Mertz BL, Manock S, et al Crotaline snake bite in the Ecuadorian Amazon: randomised double blind comparative trial of three South American polyspecific antivenoms. Bmj. 2004;329(7475):1129 Epub 2004/11/13. 10.1136/bmj.329.7475.1129 ; PubMed Central PMCID: PMCPmc527684. 15539665 PMC527684

[11] Otero R, Gutierrez JM, Nunez V, Robles A, Estrada R, Segura E, et al A randomized double-blind clinical trial of two antivenoms in patients bitten by Bothrops atrox in Colombia. The Regional Group on Antivenom Therapy Research (REGATHER). Trans R Soc Trop Med Hyg. 1996;90(6):696–700. Epub 1996/11/01. . 9015522 10.1016/s0035-9203(96)90442-3

[12] Pardal PP, Souza SM, Monteiro MR, Fan HW, Cardoso JL, Franca FO, et al Clinical trial of two antivenoms for the treatment of Bothrops and Lachesis bites in the north eastern Amazon region of Brazil. Trans R Soc Trop Med Hyg. 2004;98(1):28–42. Epub 2004/01/02. . 14702836 10.1016/s0035-9203(03)00005-1

[13] World Health Organization (WHO). WHO Guidelines for the Production, Control and Regulation of Snake Antivenom Immunoglobulins 2010:[141 p.]. Available from: http://www.who.int/bloodproducts/snake_antivenoms/snakeantivenomguideline.pdf. 10.1051/jbio/2009043 20950580

[14] Estevez J, Magana P, Chippaux JP, Vidal N, Mancilla R, Paniagua JF, et al Etude des venins des principaux serpents venimeux de Guyane française et de leur neutralisation. Bull Soc Pathol Exot. 2008;101(4):353–9. Epub 2008/10/30. . 18956820 10.3185/pathexo3174

[15] Heckmann X, Larréché S, Beneteau S, Jolivet A, Perotti F, Lehida Andi I, editors. Efficacité de l’immunothérapie dans les envenimations par Crotalinae dans l’Ouest guyanais: étude comparative sur 63 patients Première journée dédiée aux travaux scientifiques des jeunes médecins de Guyane (JDIG)—Nos internes ont du talent; 2017; Cayenne, French Guiana: Bull Soc Path Exo; 2018.

[16] Muniz EG, Maria WS, Estevao-Costa MI, Buhrnheim P, Chavez-Olortegui C. Neutralizing potency of horse antibothropic Brazilian antivenom against Bothrops snake venoms from the Amazonian rain forest. Toxicon. 2000;38(12):1859–63. Epub 2000/06/20. . 10858523 10.1016/s0041-0101(00)00082-9

[17] Otero-Patiño R, Silva-Haad JJ, Barona Acevedo MJ, Toro Castaño MF, Quintana Castillo JC, Cadavid AD, et al Accidente bothrópico en Colombia: estudio multicéntrico de la eficacia y seguridad de Antivipmyn-Tri®, un antiveneno polivalente producido en México. Iatreia. 2007;20(3):245–62.

[18] Nguyen L. Production d'immunoglobulines therapeutiques hautement purifiées (ITHP): analyse d'un procédé de purification. Biol Aujourdhui. 2010;204(1):55–9. Epub 2010/10/19. 10.1051/jbio/2009050 . 20950576

